# Countering the Modern Metabolic Disease Rampage With Ancestral Endocannabinoid System Alignment

**DOI:** 10.3389/fendo.2019.00311

**Published:** 2019-05-17

**Authors:** Ian Pepper, Aaron Vinik, Frank Lattanzio, William McPheat, Anca Dobrian

**Affiliations:** ^1^Department of Physiological Sciences, Eastern Virginia Medical School, Norfolk, VA, United States; ^2^Strelitz Diabetes Center, Eastern Virginia Medical School, Norfolk, VA, United States

**Keywords:** cannabinoids, evolution, obesity, energy balance, type 2 diabetes, NAFLD

## Abstract

When primitive vertebrates evolved from ancestral members of the animal kingdom and acquired complex locomotive and neurological toolsets, a constant supply of energy became necessary for their continued survival. To help fulfill this need, the endocannabinoid (eCB) system transformed drastically with the addition of the cannabinoid-1 receptor (CB1R) to its gene repertoire. This established an eCB/CB1R signaling mechanism responsible for governing the whole organism's energy balance, with its activation triggering a shift toward energy intake and storage in the brain and the peripheral organs (i.e., liver and adipose). Although this function was of primal importance for humans during their pre-historic existence as hunter-gatherers, it became expendable following the successive lifestyle shifts of the Agricultural and Industrial Revolutions. Modernization of the world has further increased food availability and decreased energy expenditure, thus shifting the eCB/CB1R system into a state of hyperactive deregulated signaling that contributes to the 21st century metabolic disease pandemic. Studies from the literature supporting this perspective come from a variety of disciplines, including biochemistry, human medicine, evolutionary/comparative biology, anthropology, and developmental biology. Consideration of both biological and cultural evolution justifies the design of improved pharmacological treatments for obesity and Type 2 diabetes (T2D) that focus on peripheral CB1R antagonism. Blockade of peripheral CB1Rs, which universally promote energy conservation across the vertebrate lineage, represents an evolutionary medicine strategy for clinical management of present-day metabolic disorders.

## Highlights

- Alterations in diet, exercise, and stress over the course of human cultural evolution have triggered the 21st century metabolic disease pandemic.
- CB1 receptors and their eCB ligands regulate energy balance across all vertebrate species in both the central nervous system and peripheral organs.
- Societal transitions have facilitated deregulation of peripheral eCB/CB1R system function, which subsequently induces systemic insulin and leptin resistance.
- Pharmacological blockade of peripheral CB1Rs represents a viable clinical strategy for treating the physiological complications of obesity and Type 2 diabetes.

## Introduction

While the term “pandemic” generally conjures images of a deadly virus or bacterium rapidly infecting a population, one of the most significant pandemics currently facing humanity is the global rise of diabetes and related metabolic diseases. The gravity of this crisis becomes readily apparent when examining the Diabetes Atlas released by the International Diabetes Federation (IDF). According to the 2015 Atlas, 415 million adults worldwide (1 in 11) have diabetes and nearly half of that population is presently undiagnosed (1). By 2040, the global prevalence of diabetes is expected to exceed 1 in 10, in part due to a growing number of vulnerable individuals with impaired glucose tolerance (IGT; also known as pre-diabetes). IGT facilitates the emergence of adult-onset or Type 2 diabetes (T2D), the variant accounting for 90 percent of diabetes cases in high-income nations.

The high mortality associated with T2D reflects its devastating long-term vascular complications, which can affect multiple tissues and organs such as kidneys (nephropathy), eyes (retinopathy), peripheral nerves (neuropathy), heart (cardiomyopathy), and blood vessels (atherosclerosis) (2). The persistent state of hyperglycemia (high blood sugar) in T2D patients promotes damage to the heart and blood vessels. Complex interactions between circulating glucose and the molecular pathways within endothelial and cardiac cells are believed to underlie this relationship between hyperglycemia and vascular complications (3, 4). Hyperglycemia and resistance to the glucose-clearing effects of the hormone insulin are both pathological hallmarks of diabetes. Insulin resistance demands heightened production of the hormone by pancreatic beta cells, which taxes them and leads to their eventual death. In advanced T2D patients, exogenous insulin administration is required due to insufficient capacity of their own beta cells to meet physiological insulin demand.

In addition to hyperglycemia-induced vascular complications, the devastation of the T2D pandemic stems from its inseparable connection with obesity. A meta-analysis of human clinical studies detected that obesity was associated with a 90-fold increase in diabetes risk and concluded that weight loss could be the “most important therapeutic task” for diabetics (5). A longitudinal study involving a population of overweight patients with IGT supports this conclusion, as subjects receiving an “intervention” of weight management counseling (and thus losing more weight than “controls”) reduced their risk of developing T2D by 58 percent (6). The exact relationship between T2D and obesity remains somewhat of a “chicken or the egg” controversy. In Gerald Reaven's original concept of “syndrome X” (7), which was later designated as Metabolic Syndrome (MetS) (8), insulin resistance is pre-requisite for the appearance of other MetS indications (i.e., obesity, dyslipidemia, and hypertension). While some studies on rodents (9) and humans (10) support this view, other evidence suggests that central (abdominal) obesity triggers systemic (whole-body) insulin resistance via the secretory products of adipocytes (11). Regardless of the precise cause-and-effect relationship, the coinage of the term “Diabesity” by Francine Kaufman in the title of her 2005 book (12) reflects the inherent linkage between obesity and T2D at the individual and population levels. Diabetes epidemiologist Paul Zimmet characterizes the modern “Diabesity” epidemic as a global health crisis more devastating than the Black Plague (13), and his own recent research on human populations in several geographic locations suggests intertwinement between the two conditions (14, 15). Thus, the relationship between genesis and progression of T2D and obesity can be labeled “symbiotic,” with exacerbations in one condition aggravating the other.

## Hypothesis

With the obesity-T2D symbiosis in mind, it seems therapeutically counterproductive that many of the available anti-diabetes medications (especially insulin for advanced patients) lead to weight gain rather than loss (16). As for the current generation of medications intended to promote weight loss, concerns about their psychiatric side effects remain unresolved (17). To combat the dual rise of obesity and T2D, new pharmacological interventions that synergistically benefit both conditions are needed. Even more ideal would be an agent able to tackle all aspects of MetS, as it could prevent the emergence of diabetes in vulnerable overweight individuals with IGT. In the early 2000s, Rimonabant seemed to fill the role of “transcendent” metabolic drug upon approval for therapeutic use in Europe (but not the United States). It promoted weight loss and restoration of insulin sensitivity while normalizing the blood lipid profile in several multinational clinical trials. Although Rimonabant swiftly exited the market in light of serious adverse effects, the trials provided proof-of-concept that the biological basis of Rimonabant's efficacy was translatable to human metabolic disease (18). Rimonabant's metabolic effects occurred via inverse agonism of cannabinoid-1 receptors (CB1Rs) throughout the body, including the brain. A major breakthrough in subsequent drug design efforts came from the realization that CB1Rs on peripheral organs and tissues outside the brain had their own integral contributions to the drug's clinical efficacy, with minimal contribution to its associated side effects (19). A peripherally restricted CB1R inverse agonist, modeled after a sister compound of Rimonabant (20), has demonstrated equal efficacy and reduced psychiatric liability in pre-clinical studies compared to its centrally active scaffold (21). Thus, the prospect of an all-in-one solution to MetS has not disappeared, but instead has improved while maintaining CB1R antagonism as its pharmacological backbone.

**Figure 1:**
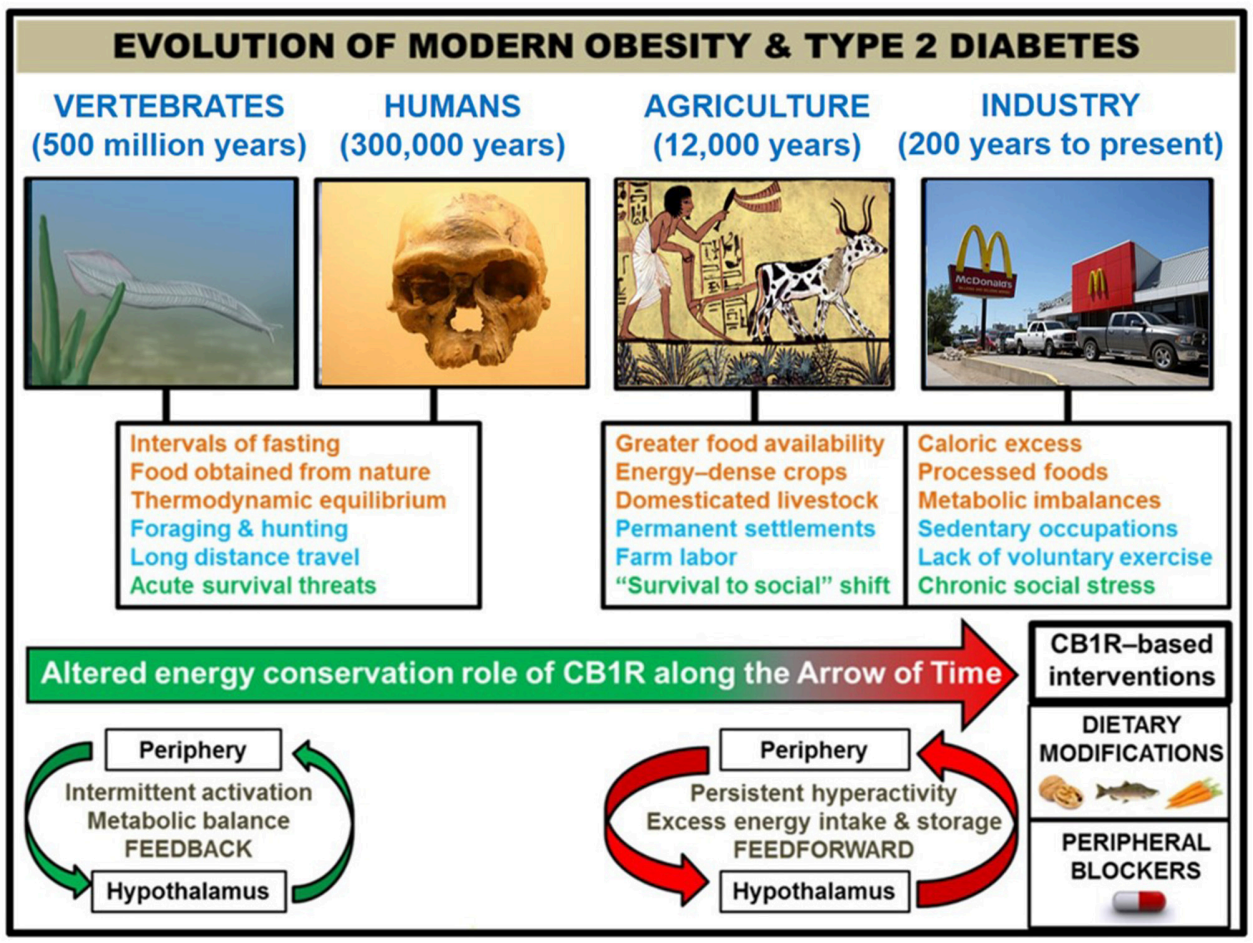
Correlation of human societal transitions with fundamental alteration of eCB/CB1R system's energy conservation function. Upon their initial appearance in the fossil record circa 300,000 years ago (22), anatomically-modern *Homo sapiens* existed in primitive hunter-gatherer societies characterized by nomadism and obtaining nourishment directly from nature. This lifestyle was likely characterized by repeated bouts of strenuous physical activity, dietary patterns which properly regulated weight at the metabolic set point, and acute stressful situations such as natural predators or extreme cold weather. The Agricultural Revolution (beginning ~12,000 years ago) eliminated many unpredictable aspects of this lifestyle that, although subject to individual variations between species, were largely preserved across 500 million years of vertebrate evolution that began with primitive chordates such as the pictured *Pikaia gracilens*. Elaborate social networks and division of labor permitted the widespread abundance of energy-dense foods from animals (e.g., livestock meat and dairy) and plants (e.g., grains and potatoes). Stress became less focused on survival and more on social situations. Although the nomadic lifestyle went largely extinct, the majority of the human population has been engaged in farm labor for much of recorded history [e.g., 90% of population during Medieval Period were rural peasants (23)]. The dominance of agriculture gave way to industrialization and urbanization starting in the 1800s, and this movement has considerably shaped the present-day living conditions within developed countries. Technological advances have dramatically altered diet (shift from natural to processed foods and metabolic imbalances like leptin/insulin resistance, skewed omega 3 to omega 6 ratio, and high glycemic load), exercise (mechanized transport and sedentary occupations removed impetus for voluntary physical activity), and stress (chronic psychosocial/emotional stressors). In order to impede human metabolic disease, it is proposed that interventions centered on the eCB/CB1R system (e.g., omega-3 fatty acid supplementation, consumption of natural product CB1R inhibitors, and a pharmacological CB1R antagonist) represent efficacious strategies grounded in the context of biological evolution. Studies of animal models and humans suggest that hyperactive CB1R signaling, maintained in and among peripheral organs and integrated by the hypothalamus, is a mechanistic culprit behind the astronomical rise in obesity/T2D since the dawn of technologically advanced societies. CB1R signaling would have functioned as an efficient regulator of energy balance and metabolism in an environment of regular physical activity and irregular, low energy density feedings. However, the introduction of a more “cushioned” lifestyle, buffered from the laws of natural selection, rendered the peripheral eCB energy integration system “vestigial” beginning in the Agricultural Revolution. It was not until widespread industrialization that the character of eCB/CB1R signaling across the brain-gut axis became a pathologically overactive feed-forward mechanism, driven by the availability of energy-dense food and lack of regular exercise.

In this paper, we will propose that dysregulated hyperactivity of CB1Rs and their parent endocannabinoid (eCB) signaling system at the molecular level drives excess energy intake and storage among individuals and the modern metabolic disease pandemic across global society. Based on evidence from vertebrate biological evolution over millions of years as well as human cultural transitions over thousands, we seek to characterize the peripheral eCB/CB1R system's functions as “vestigial” for fully developed adult humans in a modern setting of plentiful energy supply (i.e., high-calorie and/or palatable foods) obtainable with minimal physical effort. By extending this evolutionary line of thinking to therapeutics, we propose a clear rationale for the usage of peripheral CB1R blockers in the clinic. Owing to their downstream (i.e., indirect) effects on key metabolic circuits of the central nervous system (CNS), we will establish the superiority of this pharmacological approach over the central CB1R antagonist Rimonabant and current therapeutics targeting “conventional” neurotransmitters. Finally, we discuss potential new therapeutic strategies as well as currently existing ones focused on limiting peripheral CB1R activation. Our central hypothesis is that human cultural evolution has fundamentally shifted the peripheral endocannabinoid system from an energy thermostat, optimized by natural selection, to a pathologically dysregulated agent of the modern metabolic disease pandemic. Drug design efforts aimed at blocking the peripheral component of modernization-induced CB1R overactivity will pave the way for safe and effective defense against the societal infiltration of obesity and T2D (Figure 1).

## Overview of eCB/CB1R System and Timeline of Discoveries

The current picture of cannabinoid biology depicts the eCB system as a ubiquitous cellular signaling machine with an active physiological role in nearly all organs and tissues. However, formal scientific study of the eCB/CB1R system has lagged far behind humanity's firsthand observation of its effects via combustion and consumption of *Cannabis* plants (marijuana). Early indications of the eCB system's metabolic integration derived from marijuana's traditional medical usage in ancient civilizations (i.e., China and India) as an appetite inducer, although it possessed several other purposes such as pain relief. These observations of antiquity were replicated by Western medicine in “clinical trials” during the 1800s (24, 25). The sensations of hunger and euphoria classically associated with marijuana provided initial hints of its action within the CNS. Not until 1964 did the first isolation and characterization of marijuana's primary psychoactive compound tetrahydrocannabinol (THC) take place within the lab of Raphael Mechoulam (26), who also discovered the structures and *in vivo* function of the endogenous analogs to THC (i.e., eCBs) in the 1990s (27, 28). The identity of these eCB ligands' target was also determined in the 1990s via receptor binding assays of rat brain slices (29).

This newly discovered CB1 receptor became a target of interest in neuroscience, which spurred the remarkable occurrence of a “Dies Mirabilis” on March 29, 2001. Four papers from four different labs were published, all converging on the conclusion that the brain's eCB/CB1R system modulates short-term synaptic plasticity by suppressing inhibitory neurotransmission in an activity-dependent, retrograde fashion (i.e., depolarization-induced suppression of inhibition [DSI]) (30). DSI provides the framework for “canonical” eCB/CB1R signaling at the synapse. Calcium influx into a depolarized post-synaptic neuron mobilizes the eCB synthetic enzymes to produce either 2-arachidonoylglycerol (2-AG) or anandamide (AEA) from arachidonate-containing membrane lipid precursors. 2-AG and AEA are synthesized on-demand rather than stored in bulk within vesicles, and degradation by their respective metabolizing enzymes ensures that their mode of action is rapid and transient. Synthesized eCBs, after being shuttled in the reverse direction across the synaptic cleft, stimulate membrane-localized pre-synaptic CB1Rs to suppress both calcium influx and subsequent neurotransmitter release. CB1Rs are G-protein coupled receptors (GPCRs) with a seven transmembrane domain. Their activation induces numerous downstream signaling cascades, among them adenylyl cyclase inhibition and potassium (K^+^) current activation (31).

In agreement with the well-known hunger stimulation capacity of marijuana, CB1Rs exist in regions and circuits of the brain responsible for regulating food intake and reward (i.e., hypothalamus and basal ganglia). A landmark study published less than a month after the Dies Mirabilis revealed the involvement of central eCBs in the promotion of feeding behavior. It found that leptin, an adipocyte secretory factor capable of crossing the blood-brain barrier (BBB), directly opposes the actions of CB1R-mediated feeding behavior by reducing hypothalamic eCB levels (32). The rodent models of obesity used in this study had defective leptin or leptin receptor (LepR) genes, and thus demonstrated persistently elevated hypothalamic eCBs. Conversely, mice lacking CB1Rs ate less than wild-type control, and did not exhibit any anorectic response to the CB1R inverse agonist SR141716A. These findings illustrate the prominent involvement of central eCB/CB1R signaling in the modulation of processes governed by circadian rhythms. 2-AG in human serum samples (33) and AEA in rat brain samples (34) follow the same general pattern, attaining their lowest levels in the middle portion of sleep (corresponding to the midpoint of the overnight fasting period) and gradually rising in the morning upon waking. In the case of 2-AG, it was found that after reaching a peak around mid-afternoon its serum levels were directly out-of-phase with serum leptin levels (i.e., decrease in 2-AG at the same time that leptin increased). Food intake serves as the trigger for the reduction in 2-AG levels after their mid-afternoon peak. In sleep-deprived human subjects, their serum 2-AG is amplified and undergoes a diminished decline in response to meal consumption. These eCB-related changes correspond with increased sensations of appetite when sleep-restricted (35). The observed dependency of circulating eCBs on circadian rhythmicity agrees with data from hamsters demonstrating CB1R expression in their suprachiasmatic nuclei (SCN), a hypothalamic region with master control over circadian regulation (36).

Since endogenous leptin and exogenous SR141716A both induce appetite suppression, the 2001 study by Di Marzo et al. encouraged utilization of this pre-clinical candidate compound as a therapeutic for patients with metabolic diseases. As a result, SR141716A gained formal approval 5 years later within the European Union and entered the market as the anti-obesity drug Rimonabant. Early clinical trial data from across the continent generated optimism for its therapeutic potential. It delivered on its expected benefits of weight loss and reduced hyperglycemia (37), and as an added bonus it ameliorated various other cardiometabolic risk factors (i.e., abnormal serum cholesterol and triglycerides, preference for cigarettes and alcohol) among a T2D patient spectrum ranging from drug-naïve to insulin-dependent (38, 39). Upon emergence of severe psychiatric side effects such as anxiety, depression, and suicidal ideation during a trial of Rimonabant's cardiovascular efficacy (40), it abruptly vanished from the European market just 2 years after its approval. While initially a momentum-killer for the prospect of CB1R antagonist drugs, this event spurred the quest for alternative compounds able to replicate Rimonabant's clinical efficacy without its psychiatric liabilities. Re-evaluation of Rimonabant's pre-clinical data uncovered a previously underappreciated energy balancing and metabolic role for CB1Rs located on peripheral organs such as adipose tissue, skeletal muscle, liver, and pancreas (41, 42). Further studies concurred that activation of peripheral CB1Rs in multiple organs drives fat storage, insulin resistance, and other metabolic imbalances through a variety of mechanisms reviewed elsewhere (2, 43, 44). Together, this data indicated that a new class of CB1R antagonists without access to the brain could succeed where Rimonabant had failed.

**Figure 2:**
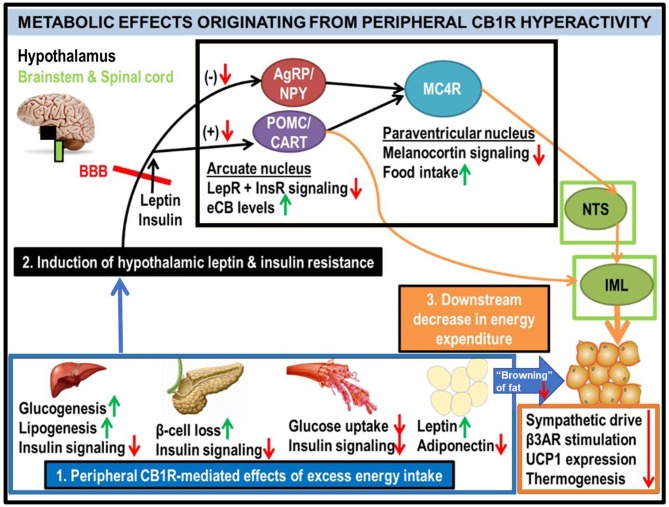
Generalized schematic of how peripheral eCB/CB1R dysfunction leads to the metabolic consequences associated with obesity and T2D. All green or red arrows indicate the observed effects of increased peripheral CB1R signaling: green corresponds to a phenotype stimulated by peripheral CB1Rs, whereas red means that the effect is inhibited. Excessive intake of energy (especially in the form of palatable high-calorie foods spawned by recent agricultural and industrial advances) fuels dysfunctional overactivity of peripheral organ eCB/CB1R signaling cascades **(1)**. This phenomenon is consistently observed in a wide range of vertebrate model species, from zebrafish to rodents, and is recapitulated in clinical studies of human subjects. The sum of deleterious metabolic consequences induced by CB1R hyperactivity within individual organs manifests as systemic resistance to the actions of both leptin and insulin **(2)**, which, respectively, are typical features of obesity and T2D. Although circulating concentrations of these hormones are often increased in patients with metabolic disorders, the resistance in both peripheral organs and the central nervous system impairs the downstream signaling cascades normally produced by these hormones' receptors (LepR and InsR). Upon crossing the blood-brain barrier (BBB), the synergistic actions of intact leptin and insulin signaling promote activation of the pro-opiomelanocortin/cocaine- and amphetamine-related transcript (POMC/CART) neurons within the hypothamalic arcuate nucleus (49). This population of neurons exists in a “tug-of-war” with the agouti-related peptide/neuropeptide Y (AgRP/NPY) neurons, as activation of one population inhibits the other. When leptin and/or insulin signaling is impaired, the activation of POMC/CART neurons is also suppressed, leading to a reduction in downstream melanocortin-4 receptor (MC4R) as well as elevation of intra-hypothalamic eCBs. Collectively, these alterations would lead to increased food intake, as MC4R activation within the paraventricular hypothalamus (PVH) promotes satiety upon arcuate LepR activation. In tandem, the reduced MC4R signaling output would hinder its transmissions to the sympathetic nervous system. Among the key affected regions are the brainstem's nucleus tract solitarius (NTS), involved in the promotion of satiety (50), as well as the spinal cord's intermediolateral column (IML), involved in energy expenditure via brown adipose tissue (BAT) thermogenesis (51–53). A convergent projection from the POMC neurons to the IML also regulates BAT activity. Together, this evidence supports the conclusion that insulin and leptin resistance resulting from peripheral CB1R dysfunction, can interfere with central pathways of energy balance **(3)**.

**Figure 3:**
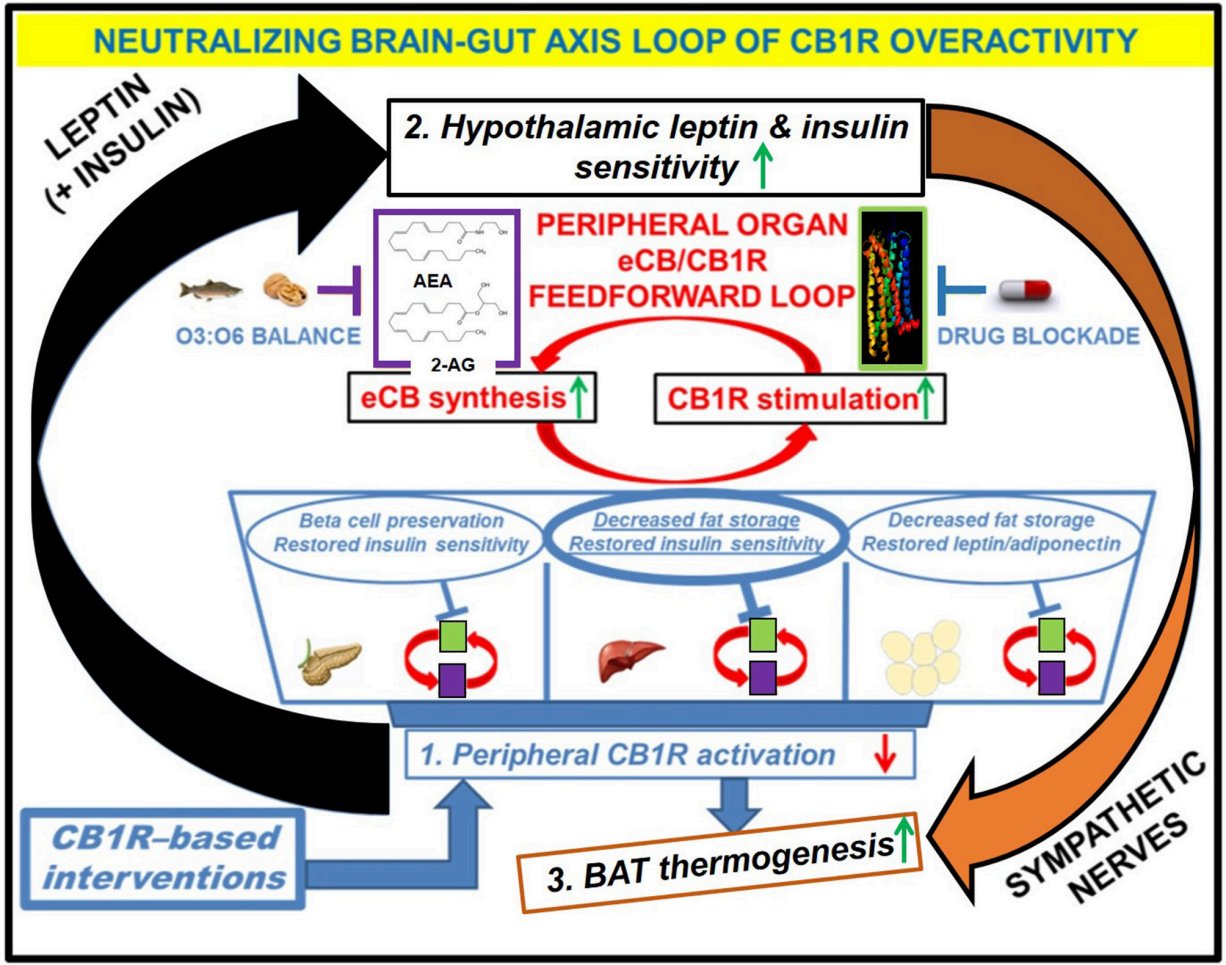
Downstream effects of peripheral CB1R blockers on the restoration of CB1R-LepR-MC4R axis homeostasis. Administration of the drug would have direct effects on the “feed-forward” loops of eCB/CB1R overactivity present in the peripheral organs of metabolic disease patients. The purple and green boxes linked cyclically by the red arrows represent a zoomed-in view of how various eCB/CB1R system-based interventions alleviate metabolic dysfunction within individual organs. The drug would directly bind to CB1Rs in peripheral tissues and prevent binding of endogenous eCBs. Increased consumption of Omega-3 fatty acids relative to Omega-6 fatty acids, either via whole foods or by supplementation, can augment the drug's efficacy via reduction of eCB ligand synthesis. The downstream effects of inhibiting the peripheral CB1R stimulation (1, blue box) would manifest as increased sensitivity to both leptin and insulin within the hypothalamus (2, black box), despite a likely reduction in the plasma levels of these hormones. Because the hypothalamus exerts top-down control over peripheral and central energy homeostasis, there would be additional downstream effects produced by alterations to the sympathetic nervous system, notably an increase in BAT thermogenesis (3, orange box). Importantly, the increased “browning” of WAT promoted by CB1R blockade (blue arrow from box 1 to box 3) would synergize with these centrally mediated effects to increase overall energy expenditure.

The gold standard of this class, identified as JD5037, epitomized the clinical objective of these “next generation” peripheral CB1R blockers. Despite not binding to brain CB1Rs as Rimonabant did, JD5037 administered to rodents with diet-induced obesity (DIO) was equally effective with respect to weight loss and other metabolic benefits (21). These effects were mediated via reversal of high leptin levels in the circulation, as antagonism of adipocyte CB1Rs diminishes DIO-induced hypersecretion of leptin. As a downstream consequence of JD5037 treatment, hypothalamic (i.e., central) LepR signaling and its anorexigenic downstream response (i.e., STAT3 phosphorylation) were also improved. These biochemical processes involved in leptin signaling are impaired in obese rodents and humans, leading to leptin resistance (45, 46). A recent follow-up study by the same group found that pharmacological silencing of central melanocortin-4 receptors (MC4Rs) completely neutralizes the beneficial effects of JD5037 but does not affect Rimonabant's efficacy (47). MC4Rs possess a downstream functional link to LepRs in the hypothalamus, as both of these receptors participate in a neural circuit that promotes energy expenditure and reduces food intake (48). Importantly, either peripheral or central CB1R activation directly opposes the effects of the central LepR-MC4R axis, promoting energy consumption and storage (Figure 2). In summary, pre-clinical studies of JD5037 confirm the hypothesis that by “evolving” Rimonabant into a peripherally restricted compound, drug safety improves while clinical efficacy remains intact. Reversal of hypothalamic leptin resistance and restoration of central CB1R-LepR-MC4R axis homeostasis both contribute to efficacy of peripheral CB1R blockade despite its lack of BBB penetration (Figure 3).

## CB1R Evolution From Primitive Animals to Human Societies

### The eCB System's Origins During Pre-vertebrate Evolution of Animals

The medical importance of CB1R's broad distribution and influence over every aspect of energy homeostasis and metabolism, from the brain's hunger signals to nutrient utilization in the periphery, is highlighted by a 2015 review article (54) which designates the eCB system as a “pivotal orchestrator of obesity and metabolic disease” due to its “multi-organ energy-stowing function.” The juxtaposition of physiological energy balancing with metabolic pathologies echoes the central theme of this paper. Modern societal transitions have transformed the human eCB/CB1R system from a functional energy preserver, aimed at preventing starvation and securing the calories necessary to power advanced physiology, to a harbinger of pathological energy storage (obesity) and glucose metabolism (T2D). The basic premise of this hypothesis mirrors that of James Neel's classic “thrifty gene” hypothesis about modern metabolic disease. This idea states that certain gene variants within human populations that maximize energy storage ability are deleterious in civilized society, despite their preservation via evolutionary selection due to the survival advantage they offered in times of scarcity and famine (55). By expanding this idea to molecular evolution on the scale of the entire animal kingdom, the eCB system was conserved over time due to the role that eCBs fulfilled as “thrifty lipids” able to influence several CNS feeding and metabolic circuits toward energy conservation (56).

Comprehension of the modern metabolic disease epidemic as a CB1R-driven phenomenon requires understanding of the eCB system's evolutionary origins. Administration of AEA to *Hydra vulgaris*, a basal animal with a “nerve net” as its primitive nervous system, modulates feeding behavior at a physiologically-relevant concentration. Furthermore, Rimonabant specifically blocks AEA's stimulation of *Hydra* mouth closure (57). This suggests that a rudimentary eCB system regulating food intake, and thus energy balance as a whole, was present in the ancestor to all presently living animals. The existence of a functional eCB system, complete with synthetic and degradation enzymes, in many other invertebrate taxa supports this view (58). Notable taxa without any observed eCB function include insects (59) and sponges (60), suggesting secondary loss of eCB system genes in these branches of life. Although several invertebrates have receptors able to bind eCBs and transduce their signals, these are not genuine CB1Rs based on sequence homology. By contrast, authentic CB1Rs exist in the genomes of all extant vertebrates, including the jawless lamprey (61).

Taken together, these comparative studies predict that an ancestral cannabinoid receptor was present in the animal kingdom's last common ancestor (LCA) which lived around 1 billion years ago (62), and underwent three distinct fates in the various lineages of animals that evolved from this LCA. The lineages giving rise to invertebrates such as *Hydra*, sea urchins, mussels, and leeches retained this ancestral eCB signaling system, whereas it disappeared from others like insects and sponges. As for the vertebrates, their initial appearance around 500 million years ago was likely accompanied by a duplication of the ancestral cannabinoid receptor (63) into CB1R and its CB2R paralog (< 50% overall identity to CB1R). The primitive eCB system of the earliest animals would have possessed all the necessary enzymes to synthesize and metabolize eCBs, as well as the putative “ancestral” CBR. It was not until the emergence of the first vertebrates that the selection pressure (i.e., increased energy demand) for the CBR to diverge into CB1R and CB2R became fully realized. The factors driving greater energy demand for vertebrates include increased nervous system complexity (64), higher metabolic cost of immune system activation (65, 66), energy-intensive foraging/predation strategies (67), and lowered growth efficiency due to their high trophic level on food webs (68). Based on invariant motifs present in all vertebrate CB1Rs but absent from invertebrate CBR orthologs or vertebrate CB2Rs, it appears that the CBR duplication event early in the vertebrate lineage optimized the eCB system's regulation of energy homeostasis.

### Conserved CB1R Structure and Function From Fish to Humans

A specific motif conserved in vertebrates and essential for proper CB1R function is the third transmembrane helical segment, which mediates eCB ligand binding and downstream signal transduction. This third helix exhibits 100% identity among human, rhesus monkey, rat, finch, newt, and pufferfish (69) but not the non-vertebrate chordate *Ciona intestinalis* (sea squirt) (70, 71). *In vivo* studies of non-mammalian vertebrates illustrate the functional implications of this structural conservation. Increased eCB levels in the goldfish brain serve as an indicator for hunger (72), just as they do within the mammalian brain. The conserved receptor function between fish and mammals also exists in the periphery, as demonstrated by the role of CB1R gene expression in the pathological accumulation of fat in adult zebrafish livers (73). In this same model organism, CB1R signaling plays an absolutely critical role in the liver's normal embryonic development (74), reflecting the diminished importance of the eCB/CB1R system's peripheral component for adults compared to juveniles. The system possesses similar importance for the development of the entire frog digestive system, from the olfactory bulb within the CNS to the organs of the gastrointestinal tract (75, 76). Starting from embryogenesis and extending into adulthood, the vertebrate eCB/CB1R system participates in a litany of conserved functions related to energy acquisition, storage, and metabolism.

Mammals utilize a unique form of temperature regulation not possessed by “cold-blooded” vertebrates such as frogs and fish. Known as brown adipose tissue (BAT) thermogenesis, it allows their bodies to generate internal heat from energy stored in BAT deposits. Interestingly, conventional white adipose tissue, which stores energy as opposed to liberating it, can differentiate into BAT (77), although in humans aging (78), and obesity (79) inhibit this process. Studies of rodents have shown that CB1Rs located on adipocytes themselves (80) as well as those expressed on forebrain and sympathetic neurons (81) act as suppressors of BAT thermogenesis. In the context of mammalian evolution, CB1Rs would have prevented complete exhaustion of white and brown adipose reserves during BAT-stimulatory conditions (e.g., prolonged cold exposure) and preserved precious power needed to maintain, among other things, the mammalian brain and its complex architecture (82, 83). Sustained energy supply was especially vital for the exponential increase in brain size among archaic hominin species, beginning with the human-chimpanzee divergence from their last common ancestor roughly 6 million years ago. Given the correlation between this transition and a reduction in overall gastrointestinal tract length (84), it is plausible that interactions between the peripheral and central eCB systems were re-configured during the evolution of humans within the hominin lineage. This scenario could potentially explain the higher energy intake and increased diet quality (i.e., animal flesh and fat sources) that coincided with brain expansion over time in the hominin lineage's members (85).

Another category of mammals with extensive need for energy reserves are hibernating mammals, which are not confined to a specific lineage but rather spread throughout the phylogeny. These organisms extensively utilize BAT when they are not actively acquiring energy during the winter months, a physiological state which would require lowered levels of eCBs within adipose tissue. BAT thermogenesis suppression serves as the final component within the “peripheral → central → peripheral” metabolic circuit of eCB/CB1R-mediated energy conservation, shared among mammals regardless of hibernation capacity and exhibited under conditions of excess energy intake (see Figure 2). In contrast with the utilization of stored fat during periods of torpor, the immediate interval before hibernation features hyperphagia (increased food consumption) and increased storage of white adipose tissue despite high circulating levels of leptin. This pre-hibernation state of “leptin resistance,” which has been directly observed and quantified in black bears (86) and brown bats (87), closely mirrors the presentation of patients with metabolic disorders. Thus, a potential circannual rhythm of the eCB system, coordinated with seasonal oscillations in leptin levels, exists in hibernating species to synchronize energy regulation with time of year (88). In line with the previously discussed literature about the eCB/CB1R system's regulation of daily circadian processes such as food intake and sleep regulation, certain lineages that evolved hibernating lifestyles have perhaps expanded this role into modulation of physiology according to the time of year.

Throughout the natural history of vertebrates, the coordinated central-peripheral crosstalk of the eCB/CB1R system conserved energy for “wild” organisms living in environments with unpredictable food availability. When humanity rapidly (on the scale of evolutionary time) civilized and successively developed agricultural and industrial technologies in order to secure a more stable and energy-dense food supply, the original selection pressures for the eCB system's evolution in vertebrates became obsolete. Despite a lack of empirical evidence regarding CB1R function in cartilaginous fish (the most basal jawed vertebrates), the liver's role as the only fat deposit available to sharks (89) for fueling their extensive migratory activity (90) suggests that liver fat storage was a major impetus for peripheral eCB/CB1R system evolution in vertebrates. With respect to metabolic disease, laboratory studies of both fish and mammals indicate that the hepatic CB1Rs are a “hotspot” shared among vertebrate species in which dysregulated activity has particularly devastating consequences. Support for this hypothesis comes from a study in which the environmental contaminant bisphenol A (BPA) upregulated eCB synthesis plus CB1R expression within the liver when administered to zebrafish. BPA-induced elevation of eCB/CB1R activity produced hepatic steatosis (i.e., buildup of fat) in zebrafish by the same mechanism of action displayed in human hepatocytes (91). Separate human studies of hepatic steatosis patients have also detected increases in both plasma AEA levels (92) and hepatic CB1R expression (93). This predicament violates the normal inverse relationship between ligand and receptor expression (i.e., greater eCB ligand expression leads to CB1R downregulation).

Perhaps the most convincing evidence for the tremendous influence of liver CB1Rs on overall metabolic dysfunction comes from mice with CB1Rs specifically deleted from the hepatocytes, but preserved in all other locations. Upon receiving high-fat diet, these conditional knockout mice did not develop numerous metabolic consequences associated with the DIO mouse model (i.e., dyslipidemia, insulin/leptin resistance, hepatic steatosis) despite gaining a typical amount of weight (94). Studies of the canine model, a mammalian species outside of the common rodent and primate models, have also corroborated the importance of metabolic eCB signaling in the liver. Two separate experiments found that Rimonabant administration to dogs with DIO significantly improved hepatic insulin sensitivity (95, 96). The combination of evidence from various animal models as well as human biomedical studies suggests that these pathological metabolic responses have deeply ingrained themselves within the vertebrate phylogeny. The strongest evidence for this deep evolutionary connection across 500 million years of vertebrate evolution exists in the positive feedback between increased eCB levels and elevated CB1R expression, demonstrated in zebrafish liver steatosis (91) and peripheral organs of obese humans (97–99) (see Figure 3).

### Modified eCB/CB1R Dynamics in Modern Society and Metabolic Disease

The constant “feedforward” loops of eCB/CB1R activity within individual organs can aggregate and form a systemic cycle that, if left unbroken, leads to progression of metabolic pathologies. The traditional “diet and exercise” remedy serves as one such circuit breaker by reducing overall eCB/CB1R system tone. In clinical intervention studies of obese and/or T2D patients, both caloric restriction and increased physical activity (alone or in combination) have produced marked improvements in cardiometabolic risk in tandem with decreased circulating eCB concentrations (100–103). Trends in circulating eCBs also recapitulate the symbiotic relationship between obesity and diabetes, as a study of post-menopausal obese women found greater plasma 2-AG levels in insulin-resistant subjects (104). High circulating eCB concentrations seen in various metabolic disease patients potentially indicate “spillover” of excess molecules synthesized by the liver and/or adipose tissue.

Zooming into the eCB/CB1R system of adipose tissue reveals a controversial picture (105), which can be resolved by considering the difference between tissue depots (i.e., visceral and subcutaneous) with respect to metabolic disease potential. Visceral adipose tissue (VAT) is more closely associated with insulin resistance and dyslipidemia as well as overall mortality (106). By contrast, subcutaneous adipose tissue (SAT) exhibits greater capacity for protective functions such as absorption of excess free fatty acids in circulation. It also supports enhanced secretion of adiponectin, a factor present in the circulation of normal-weight individuals but deficient in obese patients (107). Correspondingly, a pair of studies detected simultaneous upregulation of both eCBs as well as CB1Rs in VAT from obese MetS vs. normal-weight individuals, and the level of VAT CB1R mRNA correlated with insulin resistance, adiposity, and microvascular complications (108, 109). By comparison, one of those same studies (108) found more CB1R mRNA in SAT of normal-weight patients compared to those with MetS, and this finding falls in line with the observation of increased SAT 2-AG following 10% weight loss in an obese study population (110). The other study in the pair (109) found that in a cohort of obese patients, individuals with a CB1R gene polymorphism had 40% less VAT CB1 expression in addition to a lower prevalence of MetS and renal microvascular complications.

The theme of individual eCB system polymorphisms motivated a recent study of an ethnically and metabolically diverse Brazilian population. A polymorphism impairing expression of fatty acid amide hydrolase (FAAH), the enzyme responsible for degrading AEA, correlated with severity of both insulin resistance and obesity. This relationship with metabolic dysfunction depended on whether the polymorphism was homozygous or heterozygous, given that homozygotes displayed higher plasma AEA levels (111). Individual genetic variation of the eCB/CB1R system components, including enzymes involved in the synthesis in degradation, have emerged in various human populations as they spread themselves across the globe and formed their cultural identities starting 300,000 years ago. Although these processes have occurred on a much smaller time scale compared to the evolution of new biological species, their effect on metabolic disease potential remains significant. Another connection between eCB system function, cultural evolution, and metabolic disease derives from the imbalanced ratio of omega-3 (O3) to omega-6 (O6) fats in modern diets. Whereas, ancestral hunter-gatherers likely consumed a diet with a 1:1 ratio of these polyunsaturated fatty acids (PUFAs), this parameter approaches 1:20 or lower in the standard Western pattern diet and other modern regimens. The skewed O3:O6 ratio increases risk of obesity via elevated production of eCBs from arachidonate, an omega-6 fatty acid abundantly present in meat, dairy, and eggs as well as vegetable oils commonly used in cooking. Consuming relatively larger quantities of O3 fatty acids such as docosahexanoic acid (DHA) and eicosapentaenoic acid (EPA) effectively reduces the risk of obesity development (112).

Anthropological dissection of cultures like the Pima tribe of the Southwestern United States, whose delayed modernization directly precipitated an accelerated rise of obesity and T2D, suggests that human populations with genes optimized for energy conservation and physiological insulin resistance lost their survival advantage and became especially prone to metabolic dysfunction in a transformed environment (113). This phenomenon closely mirrors our hypothesis of the peripheral eCB system's changed role throughout human cultural evolution; thus, it seems logical to speculate that derangements in peripheral eCB/CB1R system activity manifest in these recently modernized populations. Support of this view comes from a study of obese and overweight Southwestern Native Americans demonstrating that increased levels of both eCBs in skeletal muscle [an indicator of insulin resistance based on *in vitro* experiments with human skeletal muscle cells (114)] predicted an overall trend of decreased resting energy expenditure (115). Interestingly, an earlier study comparing central eCBs among racial groups found that Native Americans had more 2-AG circulating in their cerebrospinal fluid than Caucasians (116), suggesting that heightened peripheral and/or central eCB/CB1R activity in this demographic could partially account for their increased incidence of obesity and T2D. A study of plasma eCBs in South Asians, another population with increased metabolic disease susceptibility (117), found that 2-AG levels are intrinsically elevated compared to Caucasian individuals and exhibit a greater spike in response to cold exposure (118). The clinical relevance of these findings pertains to the higher incidence of metabolic diseases among South Asians compared to Caucasians, a trend that could be associated with diminished BAT activitation due to elevated concentrations of inhibitory eCBs. Although all humans share general patterns of metabolic eCB/CB1R dysregulation with other vertebrates, specific variations among individual human populations have also emerged.

## Metabolic Therapeutics Viewed through eCB/CB1R System Evolution

Based on the peripheral eCB/CB1R mechanisms dysregulated by modern-day energy abundance, JD5037 and other pharmacological antagonists of peripheral CB1Rs represent promising clinical candidates for the treatment of obesity, T2D, and other metabolic disorders. In December 2017, the United States Food and Drug Administration (FDA) cleared JD5037 to begin Phase I clinical trials as a therapeutic for the most severe version of non-alcoholic fatty liver disease (NAFLD). NAFLD affects 75–80% of obese individuals (119) and possesses a functional link to insulin resistance since it can resolve in the presence of anti-diabetic drugs (120). Thus, JD5037 has the potential to serve as a useful therapy along with lifestyle modification for patients with the severe form of NAFLD (also called NASH for nonalcoholic steatohepatitis) by blocking the CB1R-mediated signals for hepatic lipogenesis. Since many NASH patients also have comorbid obesity, T2D, or perhaps MetS, there is an attractive possibility that JD5037 will synergistically treat these conditions. It is interesting to note that relative to Rimonabant's clinical population (i.e., obese and/or T2D patients), the scope of JD5037's initial trials is much more restricted given that NASH represents a minor subgroup of all patients with NAFLD. Although this gradual approach likely stems from the caution inspired by Rimonabant, it may also have to do with the plethora of pre-clinical evidence implicating the liver as an “epicenter” of pathological metabolic eCB/CB1R signaling. Organisms ranging from zebrafish to dogs to rodents and humans all display conserved patterns of CB1R overactivity responsible for diet-induced hepatic steatosis. This evolutionary conservation renders hepatic CB1Rs an ideal first target to achieve safe and successful pharmacological antagonism in the clinical setting.

Although the door remains open for a peripheral CB1R blocker approved for a more broad clinical population, the results of the JD5037 clinical trials will certainly shape the future of the compound class just as Rimonabant did before it. A previously underappreciated aspect of central eCB signaling, which was overlooked and disregarded by Rimonabant's manufacturer before advancing the drug to clinical trials (121), comes from its pleiotropic influence over cognitive processes outside of energy homeostasis. Early indications came from Rimonabant's observed ability to reduce dependency on alcohol and nicotine (37, 122). While initially perceived as beneficial for the reduction of overall cardiometabolic risk, this particular property ultimately foreshadowed the reason for Rimonabant's demise. Since CB1Rs in mesolimbic brain regions activate motivation and reward pathways, unrestricted silencing of these receptors will also produce characteristic anxiety and depression. Further studies in humans and other mammals have shown that mesolimbic CB1Rs also boost the mood following intense voluntary exercise (i.e., “runner's high”) (123). From the evolutionary standpoint, it is likely that the motivation and reward aspects of the central eCB/CB1R system co-evolved with the energy homeostatic aspect in order to coordinate feelings of pleasure with the successful acquisition of nourishment. As the search and pursuit of energy would have also entailed significant exposure to threats and stressors, the central eCB/CB1R system of zebrafish also influences the stress response and habituation to various stressors (124). In mammalian organisms, the eCBs of the pre-frontal cortex play a significant role in coping, resiliency, and termination of stress-activated glucocorticoid release induced by the hypothalamic-pituitary-adrenal (HPA) axis (125, 126). Thus, the central eCB system serves as one of the key mediators of the well-recognized functional overlap between regulation of energy, stress, and mood. Since evolution intertwined these functions with no room for therapeutic segregation, it is a prudent clinical strategy to consider central CB1Rs “off-limits” for the future and instead prioritize development of new peripherally restricted agents for metabolic diseases.

Many brain circuits and regions (e.g., paraventricular hypothalamus) serve as integrative hubs for both physiological energy balance and psychological stress response (127). Thus, the fact that many current anti-obesity pharmacotherapies (e.g., lorcaserin, naltrexone/bupropion, phentermine/topiramate) still target central neurotransmitters (i.e., dopamine, serotonin, norepinephrine, or a combination) remains a troubling prospect for long-term usage in weight management, especially when considering the inherent susceptibility of obese individuals to psychiatric disorders like depression (128). By restricting the field of view to the periphery, targeting the eCB/CB1R system gains a clear advantage over these other neurotransmitters. Unlike eCBs, neither dopamine nor norepinephrine has significant roles in energy regulation outside of the brain across a multitude of peripheral organs. While serotonin (5-HT) does possess ubiquity similar to the eCB system with respect to its peripheral distribution and metabolic function (129), the heterogeneity of the peripheral 5-HT receptor repertoire complicates the possibility of a comprehensive solution for obesity, T2D, or MetS via the peripheral serotonergic system. Evolutionarily, serotonin receptors have undergone multiple differentiation events within the metazoan lineage (130), unlike the single CB1R-CB2R duplication responsible for the vertebrate eCB system. Since peripheral organs possess a mixed distribution of 5-HT receptor subtypes, a pharmacological approach to metabolic disease would have to encompass multiple receptor subtypes to produce equivalent benefits to a drug exclusively targeting peripheral CB1Rs.

While peripheral CB1R blockade's efficacy seems well-established and legitimate (based on both JD5037 pre-clinical data as well as the Rimonabant clinical trials), safety concerns that have lingered ever since the cancellation of Rimonabant must be addressed. The most pressing issue stems from the unacceptable psychiatric side effects, which have seemingly been resolved by the non-brain penetrant design of JD5037. Whereas acute treatment of mice with Rimonabant produces noticeable anxiety in a behavioral assay known as the elevated plus maze (EPM), treatment with JD5037 does not alter EPM performance relative to vehicle-treated controls (21). Additionally, another compound in the peripheral CB1R inverse agonist class (TM38837) displays significantly lowered potential to promote fear responses in mice compared to Rimonabant (131). Nonetheless, the lack of obvious behavioral effects in a mouse model does not guarantee complete safety of the drug for human patients. Perhaps the most relevant concern for the peripheral CB1R antagonists derives from the controversial literature regarding CB1R function on peripheral nerve terminals. It has already been mentioned that neuropathy (especially in the extremities) is a major complication of diabetes. In an *in vitro* study of differentiated neurons, pharmacological activation of CB1Rs was able to counteract the high glucose-induced impairment of neurite outgrowth (132). The high-affinity CB1R agonist WIN 55,212-2 was also able to relieve mechanical allodynia (i.e., pain sensation) when applied to *in vivo* rat models of both type 1 and type 2 diabetes (133). These findings do fit in with the established analgesic properties of central CB1R activation. However, they are confounded by other studies of diabetic rodents that reach the exact opposite conclusion: CB1R antagonism is able to rescue peripheral nerve degeneration and alleviate the inflammation and oxidative stress associated with diabetic nerve injury (134, 135). With respect to the CNS, the increased sympathetic outflow resulting from improved central leptin and melanocortin signaling should also be monitored. This vigilance will ensure that the adverse effects plaguing MC4R agonists when used as anti-obesity drugs (e.g., heightened blood pressure) do not appear.

As was observed in the JD5037 pre-clinical studies, blockade of peripheral CB1Rs indirectly leads to restoration of impaired LepR and MC4R signaling in the hypothalamus, indicating that a drug unable to enter the brain can still affect key brain circuits involved in metabolism. This homeostatic restoration of the CB1R-LepR-MC4R axis at both the central and peripheral levels bears remarkable similarity to the molecular changes induced by a bariatric surgical procedure known as a sleeve gastrectomy, which essentially shrinks the stomach. The procedure performed on rats resulted in significant downregulation of CB1R in the stomach (peripheral) and the hypothalamus (central) at day 90 of the post-surgery period, with reversal of leptin resistance and a dramatic increase in hypothalamic MC4Rs accompanying these CB1R expression changes (136). The blockade of peripheral CB1Rs and sleeve gastrectomy lead to metabolic improvements and lower body weight maintenance through a common molecular mechanism, leading to the exciting possibility that peripheral CB1R blockers will one day secure a niche as adjuvant medication for bariatric surgery participants.

CB1Rs of the digestive tract, including tongue, stomach, and intestines, have not been previously discussed in this work, but are reviewed extensively elsewhere (56, 137). In line with the energy conservation role of peripheral CB1Rs, these receptors play a role in sensing energy-dense food (i.e., high in fat and/or sugar) and, via connections to the mesolimbic circuitry, increase pleasurable feelings associated with its consumption. This type of energy-dense (but nutrient-poor) food intake is characteristic of the so-called Western pattern diet adopted by many developed countries, and often occurs despite no urgent sensation of hunger (i.e. “snacking”). Thus, peripheral eCBs may also possess the ability to modulate abnormal patterns of hedonic (pleasure-seeking) feeding behavior in addition to its well-established capacity to regulate homeostatic (energy-balancing) feeding, which presents a tantalizing possibility for attacking the “Diabesity” epidemic on two fronts. A recent study explored this possibility by examining DIO mice given a Western Diet (WD) high in both fat and sugar rather than a standard HFD. Due to increased amount and rate of food consumption, the WD-fed mice were obese and had elevated eCBs in the plasma and small intestine compared to those fed normal chow. Blockade of peripheral CB1Rs effectively undercut the development of obesity by reducing the WD-associated hyperphagia, and importantly it did not affect consumption in the mice fed normal chow (138). These results corroborate an earlier study utilizing the non-human primate model of marmosets, in which Rimonabant administration selectively reduced intake of sweet food (139). Thus, there is legitimate evidence to suggest that peripheral CB1R blockers could synergistically reduce the palatability of “junk food” in order to augment their efficacy as agents of metabolic improvement.

Due to the ubiquity of CB1Rs in the periphery, there exists an intriguing possibility of having a CB1R antagonist compound “library” able to tackle a variety of metabolic ailments. A chief priority for this agenda is the design of a liver-specific blocker, given the evolutionary conservation of hepatic CB1R function and dysfunction. A possible approach for delivering targeted effects to the liver utilizes prodrugs that only liver enzymes can metabolize to active form (140). Exploitation of a truncated CB1R transcriptional isoform known as CB1b, which has restricted tissue distribution compared to full-length receptor, could also lead to a liver-directed agent with potential auxiliary targets. Detailed single-cell dissection experiments revealed that CB1b was expressed in human hepatocytes and pancreatic β-cells, but not the brain or skeletal muscle (141). An earlier study of the human-specific CB1R isoforms found no CB1b in the liver despite detecting CB1b in the brain (142). However, there was a noticeable decline in central CB1b expression from fetal life to adulthood, and its levels in the adult brain are much lower compared to the standard CB1R or the CB1a splice variant. The disparities between the two studies can be reconciled by acknowledging their different methodologies and patient samples. In particular, the more recent work (141) sorted subjects by BMI and detected significantly more CB1b isoform in liver and pancreas samples from patients with a BMI ≥30 vs. those with BMI < 30. When combined with the older study's detection of CB1b expression in human adipose tissue, this suggests that CB1b acts as an “obesity-upregulated” isoform: normally dormant in lean patients but transcriptionally induced in peripheral tissues of obese individuals. If correct, the CB1b isoform would become a highly coveted therapeutic destination for metabolic disease. The specificity of CB1b's peripheral distribution, along with its relatively low (or perhaps absent) CNS expression would decrease the risk of BBB penetration and any potential psychiatric liabilities. Intriguingly, receptor-binding assays indicate that the CB1b to CB1R affinity ratio is significantly greater for JD5037 than for Rimonabant (141).

Conscious changes to the diet aimed at reducing endogenous eCB levels can also produce therapeutic benefits. One of the most vital and easily implementable dietary modifications would consist of improving O3:O6 ratio via an omega-3 supplement such as krill oil. When provided to an obese and overweight cohort over the course of 24 weeks, it simultaneously reduced eCBs and triglycerides in circulation (143). Since fish oil administration was less effective than krill oil at reducing plasma eCBs in an earlier intervention study (144), the precise ratio of omega-3 fats (i.e., EPA vs. DHA) likely also influences eCB tone and metabolic syndrome parameters. Outside of omega-3 supplementation, the reduced intake of animal products high in arachidonate, combined with increased intake of plant products with phytochemicals able to act as natural CB1R antagonists (i.e., falcarinol in carrots and ginseng), could also help neutralize pathological eCB/CB1R dysfunction induced by the typical Western Diet (145). While the influence of exercise and stress on peripheral eCB/CB1R function have been less thoroughly examined in human subjects, pre-clinical studies indicate that both physical inactivity and excessive psychosocial stress can exacerbate diet-induced eCB/CB1R dysregulation. Compared to sedentary controls, obese rats subjected to a consistent regimen of endurance exercise experienced significant reductions in adipocyte size accompanied by lowered local CB1R expression (146). Another rat study found that exercise reversed HFD-induced increases in skeletal muscle AEA levels (147). In a mouse model of persistent stress exposure, the excessive glucocorticoid release caused by corticosterone-treated drinking water triggered downstream emergence of metabolic syndrome via a mechanism characterized by peripheral eCB hyperactivity (148). A holistic view of the lifestyle factors contributing to obesity and metabolic disease (i.e., diet, exercise, stress) allows for full comprehension of the peripheral eCB system's tremendous influence over pathogenesis, as well as its response to various non-pharmacological “soft” interventions.

The recently emerging field of eCB/CB1R developmental biology reveals a particular plasticity of this system in response to maternal energy status (i.e., food intake and obesity). This phenomenon warrants consideration when selecting the ideal metabolic interventions for certain individuals. Experimental evidence from rodents demonstrates that either maternal food deprivation (149) or obesity (150) can induce sex-specific alterations in expression of CB1R as well as other eCB system components (i.e., synthetic and degradative enzymes) both peripherally and centrally. These changes are functionally linked to offspring adiposity, energy expenditure and food preference (151, 152). Additionally, a baboon model demonstrates that the eCBs of maternal and placental adipose tissue exhibit communication with each other (153), and that maternal HFD actually decreases circulating eCBs in the fetal liver, thus promoting hepatic apoptosis (154). In both mouse (155) and primate (156) models, other experiments demonstrate the reversibility of maternal HFD-induced offspring metabolic defects via increased O3:O6 ratio in maternal tissue or plasma. Thus, during development the peripheral eCB system acts as a master programmer of compensatory metabolic responses to suboptimal maternal nutrition. This evolutionarily conserved role of the peripheral eCB/CB1R system, in combination with its instrumental duties in organizing the fetal pancreatic microarchitecture (157) and stimulating the newborn suckling response via AEA in breast milk (158), confirms that our hypothesis of the peripheral eCB/CB1R system's modern “vestigiality” should be qualified to apply only to adults or post-adolescent individuals. For newborns and children, individuals with prominent metabolic demands for their proper growth and development, depletion of eCB signaling could have deleterious effects. The observed maternal programming of the fetal eCB/CB1R system in a variety of mammals supports this idea, meaning that the growing childhood obesity crisis will require an alternative solution outside of peripheral CB1R antagonism.

## Conclusions and Future Directions

In summary, this paper postulates that the rapid transitions from hunter-gatherer to agriculturalist and finally industrialist society has led to the modern crisis of metabolic disease via hyperactivity of the CB1Rs originally intended to promote survival. Peripheral CB1R blockade represents a novel “evolutionary medicine” strategy for eliminating a root cause of the epidemic. We have integrated evidence from across many scientific disciplines to suggest that in obese adult humans, peripheral eCB system overactivity represents an evolutionary “mismatch” accessible to pharmacological agents (and modifiable by various lifestyle interventions) due to its focused role in energy homeostasis. By contrast, we have utilized the same evolutionary logic to demonstrate that current approaches cannot safely target central CB1Rs due to their pleiotropic functions in stress and mood regulation that emerged during vertebrate evolution.

**Figure 4:**
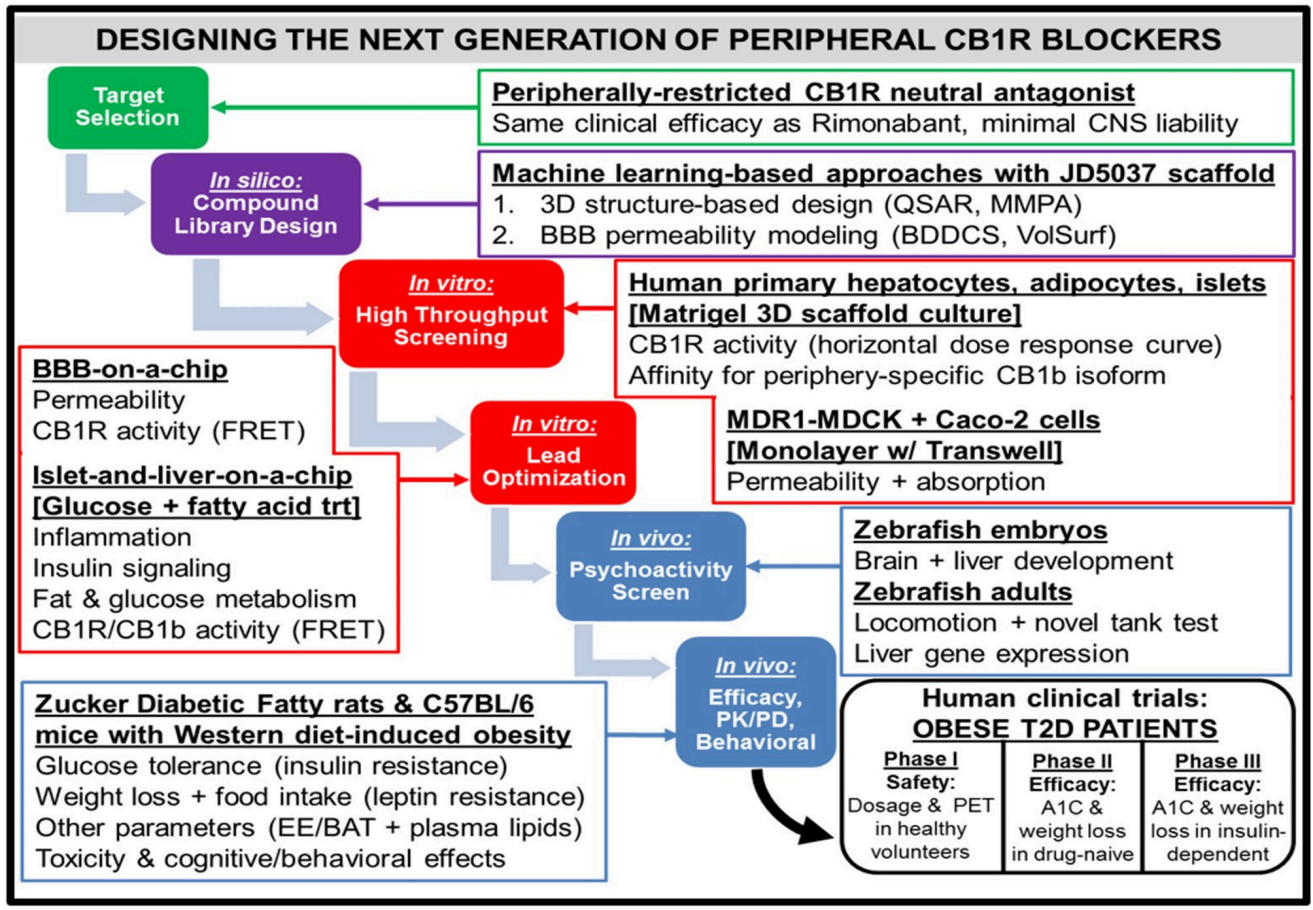
Workflow for the design of a hypothetical “next-generation” peripheral CB1R blocker. At all phases of the design process, efficacy (i.e., metabolic effects on peripheral organs) as well as safety [i.e., impermeability to blood-brain barrier (BBB)] will receive equal emphasis. Computerized techniques such as quantitative structure-activity relationship (QSAR) modeling and matched molecular pair analysis (MMPA) will help identify potential leads by utilizing scaffolds of existing CB1R inhibitor compounds (e.g., JD5037). In parallel, these leads will be subjected to the sorting scheme of Biopharmaceutical Drug Disposition and Classification System (BDDCS) (159) in order to filter out compounds with undesirable BBB penetrability. Two successive *in vitro* screening procedures will be utilized on the remaining lead compounds. The first will consist of a high-throughput screen, using primary cells from relevant peripheral tissues to characterize efficacy and Transwell permeability assays to characterize safety. Importantly, the desired activity profile for a neutral antagonist will consist of a horizontal dose-response curve in which all concentrations of drug lead to CB1R activity unchanged from baseline levels. The second *in vitro* screen will optimize the lead candidates, using more sophisticated 3-D organoid models that closely mimic human physiological conditions. To ensure precise quantification of each lead compound's CB1R activity as well as imaging of each compound's receptor binding affinity for the receptor, newly developed CB1R biosensors based on fluorescence resonance energy transfer (FRET) (160) can be incorporated into the procedure. The two-step design will also guide the approach to pre-clinical testing of the potential compounds *in vivo*. Starting with an initial “medium-throughput” screen using zebrafish embryos and adults, the non-psychoactive compounds that demonstrate high efficacy will be administered to rodents. Both rats and mice with metabolic dysfunction will be utilized, and the mice will be given a high-fat and high-sugar Western diet as opposed to the traditional high-fat diet used to induce pathology. The compound that emerges victorious from this final phase will be submitted for FDA approval as a general agent of metabolic improvement, which could potentially lead to an official designation as a therapeutic for Metabolic Syndrome. The trial population will focus on obese patients with Type 2 diabetes, and thus will have primary endpoints of both glycated hemoglobin (A1C) and weight loss over time. A potential advancement for the conduction of these clinical trials will be the emergence of imaging techniques for CB1R binding in peripheral tissue (161), similar to PET scans for the brain.

As previously mentioned, JD5037's current limited clinical niche still leaves the door open for a first-in-class peripheral CB1R blocker indicated for general improvement of all parameters associated with Metabolic Syndrome. Considering that no FDA-approved drugs currently exist for treatment of either MetS or IGT (pre-diabetes), a peripheral CB1R blocker able to fulfill this role will prove instrumentally useful as a “preventative medicine” approach to reducing the global metabolic disease burden. Figure 4 outlines a hypothetical drug discovery approach for developing a next generation peripheral CB1R blocker, grounded in utilization of the latest discoveries in cannabinoid research. The insights featured in this plan include the recently elucidated 3D crystal structure of the human CB1R (162) and the use of zebrafish as a medium-throughput *in vivo* screening option due to their eCB/CB1R system's similarity to its human counterpart. The development of CB1R blockers with a neutral antagonist profile (i.e., maintains equilibrium between CB1R activity and inactivity; compare to inverse agonist which shifts CB1Rs to inactive state) could provide distinct safety advantages over inverse agonists such as JD5037 while still preserving efficacy (163). In support of this idea, the CB1R neutral antagonist AM6545 was able to comprehensively restore metabolic function in a mouse model of obesity induced by monosodium glutamate (164). This extra layer of security offered by neutral antagonism could be of special relevance to those obese individuals with severe comorbid depression, as even residual amounts of CB1R inverse agonist crossing the BBB could have psychiatric consequences. An important potential caveat regarding the behavioral and BBB permeability testing in JD5037's pre-clinical studies is that they were conducted on lean rather than obese animal models. Similarly, a pilot study of the alternative peripheral CB1R antagonist TM38837's safety demonstrated that it did not produce central effects in human subjects, but this was conducted on healthy individuals instead of ones with metabolic disease (165). Disruptions to the blood-brain barrier are characteristic of obesity, including reduced expression of tight junction proteins that maintain BBB integrity (166). Thus, peripheral CB1R blockers should undergo further pre-clinical testing of BBB permeability with obese animal models to ensure that their peripheral restriction persists in human clinical trials, even if the BBB itself becomes compromised.

The phrase “leave no stone unturned” should apply to all future basic and clinical research on the eCB/CB1R system. This paper has certainly attempted a comprehensive incorporation of new findings and ideas into the established concept of CB1R hyperactivity driving metabolic disease. However, it has only scratched the surface of the complexity involved in this pathological relationship. One of the most basic unanswered questions in all of cannabinoid biology remains mostly unanswered, especially with respect to non-neuronal signaling (167): Why are there two eCB ligands instead of one? Cracking this fundamental enigma will likely have a ripple effect in terms of devising accurate biomarkers of eCB/CB1R dysfunction that correlate with obesity and metabolic dysfunction, as the source(s) and effects of circulating eCBs also remain somewhat mysterious (168). From the perspective of social and cultural evolution, more work will need to be done to understand why chronic cannabis users (a rising population in modern times) seem to exhibit lower risk of obesity and T2D (169) despite perpetual exogenous stimulation of CB1Rs. An answer can perhaps come from consideration that non-psychoactive cannabidiol (another cannabinoid compound present in marijuana) has been shown to attenuate hepatic steatosis, thus counteracting the deleterious metabolic effects of CB1R stimulation by THC (170). Other pharmacologically relevant factors which are intriguing but require further characterization include a peripheral circadian rhythm of CB1R expression in the liver (171), and the effects of CB1R blockade on reducing systemic obesity-associated inflammation via transformation of the gut microbiome (172, 173). In the meantime, the near future should bring a new crop of peripheral CB1R blockers vying for the chance to erase the memory of Rimonabant's debacle and accelerate interest in this pharmacological niche. An appreciation of the endocannabinoid system's very beginnings enhances both our intrinsic curiosity and our guidance toward its molecular deconstruction. Such a research agenda, if fruitful, will eventually give rise to a shining exhibition of human ingenuity—after thousands of years unknowingly using a CB1R agonist from nature as medicine, humanity has a chance to develop its own synthetic “polar opposite” and save itself from the unrelenting metabolic disease rampage.

## Author Contributions

IP conceived and wrote the manuscript. AV, WM, FL, and AD critically reviewed the manuscript.

### Conflict of Interest Statement

The authors declare that the research was conducted in the absence of any commercial or financial relationships that could be construed as a potential conflict of interest.
